# Protein Loss Enteropathy as an Initial Presentation of Gastric Epstein–Barr Virus Lymphoma

**DOI:** 10.1155/2022/5143760

**Published:** 2022-06-10

**Authors:** Bilal Niazi, Saad Ali, Sameh Elias, Michael Sciarra

**Affiliations:** ^1^Department of Internal Medicine, HMH-Palisades Medical Center, North Bergen, NJ, USA; ^2^Department of Gastroenterology, HMH-Palisades Medical Center, North Bergen, NJ, USA

## Abstract

Protein loss enteropathy (PLE) is a complex disease process that can result in potentially fatal protein losses. Gastrointestinal protein losses usually arise from damage to the gastrointestinal mucosa or from lymphatic obstruction. The goal of management is to identify and treat the underlying causes and maintain normal serum protein levels. Here, we present a patient with diarrhea and generalized edema, with decreased serum albumin and gamma-globulin levels, concerning for protein loss enteropathy. He was ultimately found to be positive for HIV infection, and his stool alpha-1 antitrypsin levels were diagnostic of protein loss enteropathy. His endoscopic and histologic evaluation revealed gastric Epstein–Barr virus-encoded small RNA- (EBER-) positive lymphoma. Though gastrointestinal lymphomas are known to cause PLE, this will be the first documented case of EBER-positive gastric lymphoma presenting with PLE. We hope to bring awareness to this unique presentation to aid in expedient diagnosis and treatment to avoid delays in treatment and potentially fatal outcomes.

## 1. Introduction

Diffuse edema and hypoalbuminemia are common initial presenting symptoms in protein loss enteropathy (PLE). PLE is a complicated and rare disease process that does not have a well-known incidence or prevalence [1]. PLE is usually due to gastrointestinal mucosal injuries or lymphatic obstructions. Mucosal injuries can occur from inflammatory bowel disease, infectious etiologies, or autoimmune causes [2]. Lymphatic obstruction can occur due to lymphangiectasias or lymphomas [2]. Risk factors for gastrointestinal lymphomas include immunodeficiency disorders such as HIV infection and other infections such as Epstein–Barr virus (EBV) and human herpesvirus-8 (HHV8) [3]. Here, we present a unique case of Epstein–Barr virus-encoded small RNA- (EBER-) positive lymphoma presenting as protein loss enteropathy in the setting of newly diagnosed HIV infection. This will be the first documented case of EBER-positive lymphoma presenting with PLE. We hope to bring awareness to this unique presentation of EBER-positive gastrointestinal lymphoma.

## 2. Case Report

We present a 38-year-old male presenting to the emergency department for generalized weakness, diarrhea, and leg swelling for one month. He was an immigrant and reported sexual encounters with multiple other males for many years. He had no known medical or surgical history. He denied tobacco or illicit drug use and only drank alcohol occasionally.

**Figure 1:**
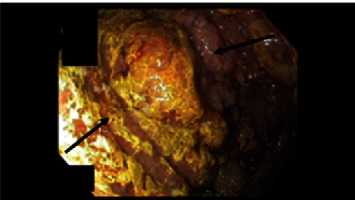
Esophagogastroduodenoscopy of the gastric mucosa reveals a suspected mass in the gastric mucosa, as highlighted by arrows, along with areas of inflammation. Imaging is limited by bile reflux. Image Credit: Michael Sciarra.

**Figure 2:**
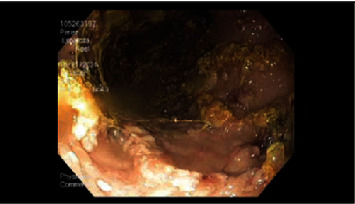
Esophagogastroduodenoscopy of gastric mucosa. The gastric body reveals active areas of inflammation. Image Credit: MichaelSciarra.

**Figure 3:**
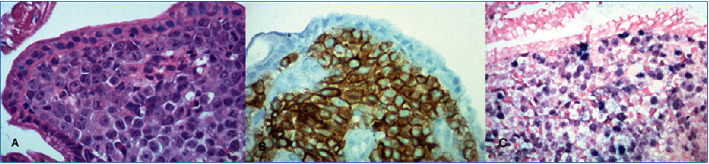
Histological evaluation of gastric mucosa. Slide (a): High-grade lymphoma seen under the gastric surface epithelium. Slide (b): Positive reactivity for CD30 with the use immunostaining. Slide (c): Tumor cells found to be positive for Epstein–Barr virus-encoded small RNA (EBER) with in situ hybridization. Image Credit: Kunchang Song.

On initial examination, his vitals were unremarkable, though his physical exam was remarkable for cachexia and bilateral lower extremity edema. No oral thrush, rales/rhonchi, or skin lesions were noted. Initial lab work was significant for hypoalbuminemia <1.5 and positive HIV-1 antibody testing with a CD4 count of 104. Additionally, protein electrophoresis revealed decreased serum gamma globulins levels (0.8 g/dL) and increased stool alpha-1 antitrypsin clearance (695 mg/L). Stool cultures were positive for giardia lamblia infection. No evidence of pulmonary pathology was noted on chest X-ray. CT imaging of his chest, abdomen, and pelvis revealed scattered enlarged lymph nodes isolated to under his diaphragm. He was started on intravenous metronidazole, 30 grams of supplementary protein, and 190 grams of medium-chain triglycerides (MCT) daily for protein loss enteropathy (PLE) suspected to be due to giardia lamblia infection. His diarrhea progressively resolved by day 4 of metronidazole treatment without improvement of his serum albumin levels. The trend of serum albumin levels is given in Table 1. Subsequently, esophagogastroduodenoscopy was performed, which revealed a gastric mass with gastritis, as shown in Figure 1 and Figure 2. A gastric biopsy revealed immunophenotypic features consistent with primary effusion type high-grade Epstein–Barr virus- (EBV-) related CD30+ lymphoma, as shown in Figure 3. The proliferative Ki-67 index was found to be high at 99%.

His protein supplementation was progressively increased to 96 grams of protein daily without improvement of his albumin levels to any greater than 2.6. His hospital course was further complicated by deteriorating mental status, renal failure requiring hemodialysis, and acute liver failure. Initially, a chemotherapy regimen of etoposide, prednisone, vincristine, cyclophosphamide, doxorubicin, and rituximab was recommended for lymphoma treatment but ultimately held due to multiorgan failure. Ultimately, a decision was made to place the patient into hospice care, and he expired within 24 hours of hospice admission.

## 3. Discussion/Conclusion

Protein loss enteropathy is a complex disease process that can occur from insults to gastrointestinal mucosa and lymphatic obstruction. Mucosal erosions can occur from inflammatory bowel disease, intestinal tuberculosis, hypertrophic gastritis, eosinophilic gastritis, systemic lupus erythematosus, and infections such as *Giardia lamblia*, *Clostridium*, *Campylobacter*, *Salmonella*, and *Rotavirus* [2]. In comparison, lymphatic insults can occur from lymphangitis, lymphomas, lymphodysplasia, sarcoidosis, congestive heart failure, or restrictive pericarditis [2]. Protein loss enteropathy commonly presents with weight loss and generalized edema and is suspected in patients with low serum albumin and gamma globulin levels. It is confirmed with stool analysis revealing increased alpha-1 antitrypsin clearance levels [1, 4]. Our patient presented with weight loss, generalized edema, low serum albumin and gamma globulin levels, and an elevated alpha-1 antitrypsin stool clearance consistent with protein loss enteropathy.

PLE is managed by identifying the underlying etiology and initiating the appropriate treatment and nutritional supplementation with protein and medium-chain triglycerides [1]. Our patient was found to have a giardia lamblia infection and started on intravenous metronidazole. Colonoscopy was deferred as there was a likely clear etiology for his diarrhea. Additionally, his diet was supplemented with appropriate protein and MCT. His diarrhea resolved with metronidazole treatment which suggested resolution of giardia lamblia infection [5]. Despite the resolution of giardia lamblia infection and appropriate nutritional support, his albumin levels remained persistently low, suggesting an alternative etiology of PLE. Subsequently, esophagogastroduodenoscopy with biopsy revealed gastric EBER-positive lymphoma.

Primary gastrointestinal tract lymphomas are a known etiology of PLE and usually present with nausea, vomiting, weight loss, and even obstruction [6]. They represent <1% of all gastrointestinal tumors [7]. Risk factors for gastrointestinal lymphomas include celiac disease, immunosuppression, and infections such as *Helicobacter pylori* and Epstein–Barr virus [8, 9]. Our patient presented with weight loss and newly diagnosed HIV infection, consistent with known presentation and risk factors for lymphoma. Furthermore, his diagnosis was confirmed with the immunohistological evaluation of gastric mucosa, revealing EBER-positive lymphoma, consistent with stage II disease based on CT findings. PLE has not been previously described as a presentation of EBER-positive gastric lymphoma.

Epstein–Barr virus-encoded small RNA positivity has implicated a worse prognosis in certain lymphomas [10]. The underlying immunocompromised state of HIV infection leads to increased risk of Epstein–Barr virus. Epstein–Barr virus is known to have oncological properties and often associated with primary effusion lymphoma, as seen with our patient [11]. Diagnosis of the EBER-positive lymphoma subtype is made histologically with in situ hybridization of Epstein–Barr virus-encoded small RNA, and staging is established by the extent of lymph node involvement [12]. Treatment of EBER-positive lymphoma in the setting of HIV is primarily targeted with chemotherapy and HAART therapy for underlying HIV infection [11]. HIV infection, in particular, is associated with a 60–200 fold increased relative risk in non-Hodgkin lymphomas such as EBER-positive lymphomas [13]. Interestingly, HIV itself does not appear to carry oncologic transformation properties, and in vitro southern blot analyses of HIV-associated lymphomas have failed to detect HIV genes [14]. This suggests that the immunocompromised state and underlying oncologic properties of the infectious source play a more significant role in the progression of lymphomas rather than HIV infection itself.

The pathophysiology of PLE from lymphoma is poorly understood. As albumin is a water-soluble molecule, it diffuses freely across the gut interstitium against a pressure gradient in the intestinal lumen [2]. It is a valuable marker of protein loss as it has a slower turnover than other proteins and is the most affected protein [15]. In healthy people, enteric protein loss amounts to 1-2% of entire protein stores and less than 10% of albumin stores [16]. However, lymphoma can obstruct the lymphatic system, thereby disrupting the diffusion gradient of albumin from the intestinal lumen into the gut interstitium leading to enteric losses of protein.

Protein loss enteropathy is a rare disease complex that presents clinically with diarrhea, weight loss, and generalized edema. It is usually due to underlying pathology in gastrointestinal mucosa and lymphatic obstructions. Gastrointestinal lymphomas should be expediently ruled out as they remain a potentially fatal cause of PLE. PLE has not been previously described as an initial presentation of Epstein–Barr virus-encoded small RNA-positive gastric lymphomas. We hope that our unique case will bring awareness of PLE as an initial presentation for EBER-positive lymphoma.

## Figures and Tables

**Table 1:** The plateau of serum albumin levels.

	Hospital day 1	Hospital day 6	Hospital day 12	Hospital day 18	Hospital day 24	Hospital day 30	Hospital day 36 (death)
Albumin (g/dL)	<1.5	<1.5	1.7	1.9	1.9	2.6	2.1

## Data Availability

The data used to support the findings of this study are included in references.
